# A prospective study of HLA*B-5801 genotyping in preventing allopurinol- induced severe cutaneous adverse reactions

**DOI:** 10.1186/2045-7022-4-S3-O4

**Published:** 2014-07-18

**Authors:** Tai-Ming Ko, Jer-Yuarn Wu, Yuan-Tsong Chen, Chen-Yang Shen

**Affiliations:** 1 Institute of Biomedical Sciences, Academia Sinica, Taipei, Taiwan, Taiwan; 2 Academia Sinica, Institute of Biomedical Sciences, Taiwan

## Background

Allopurinol, a commonly prescribed medication for gout and hyperuricemia, is a frequent cause of severe cutaneous adverse reactions (SCAR), which include the hypersensitivity syndrome, Stevens-Johnson syndrome, and toxic epidermal necrolysis. Allopurinol-induced SCAR is strongly associated with the HLA-B*58:01 allele. We sought to prevent allopurinol-induced SCAR by using HLA-B*58:01 screening to prospectively identify subjects at risk for this condition.

## Methods

From 14 hospitals in Taiwan, we recruited 2037 candidate subjects who had indication for allopurinol treatment but had not taken allopurinol previously. We genotyped DNA purified from the subjects' PBMC to determine whether they carried the HLA-B*58:01 allele. Those testing positive for HLA-B*58:01 (20.57% of the total) were advised not to take allopurinol and were given an alternative medication or advised to continue taking their pre-study medication; those testing negative (79.43%) were given allopurinol. We interviewed the subjects by telephone once a week for 3 months to monitor them for symptoms. We used the estimated historical incidence of SCAR for comparison.

## Results

Mild, transient rash developed in 4.63% of subjects during follow-up; more widespread rash was observed in 0.13% of subjects, who were hospitalized. SCAR did not develop in any of the HLA-B*58:01-negative subjects receiving allopurinol; this is in contrast to the 4 expected cases of SCAR based on the estimated historical incidence of allopurinol-induced SCAR (0.29%) (p value=0.0266; Fisher's two-tailed exact tests).

## Conclusions

The identification of subjects carrying the HLA-B*58:01 allele and the avoidance of allopurinol therapy in these subjects were strongly associated with a decreased incidence of allopurinol-induced SCAR.

## Funding

Funded by the Academia Sinica Genomic Medicine Multicenter Study and National Science Council of Taiwan.

